# Evaluation of Length of Stay, Care Volume, In-Hospital Mortality, and Emergency Readmission Rate Associated With Use of Diagnosis-Related Groups for Internal Resource Allocation in Public Hospitals in Hong Kong

**DOI:** 10.1001/jamanetworkopen.2021.45685

**Published:** 2022-02-04

**Authors:** Yushan Wu, Hong Fung, Ho-Man Shum, Shi Zhao, Eliza Lai-Yi Wong, Ka-Chun Chong, Chi-Tim Hung, Eng-Kiong Yeoh

**Affiliations:** 1 Jockey Club School of Public Health and Primary Care, The Chinese University of Hong Kong, Hong Kong Special Administrative Region, Hong Kong, China; 2 Centre for Health Systems & Policy Research, The Chinese University of Hong Kong, Hong Kong Special Administrative Region, Hong Kong, China; 3 Chinese University of Hong Kong Medical Centre, Hong Kong Special Administrative Region, Hong Kong, China; 4 Shenzhen Research Institute, The Chinese University of Hong Kong, Shenzhen, China

## Abstract

**Importance:**

Hong Kong’s internal resource allocation system for public inpatient care changed from a global budget system to one based on diagnosis-related groups (DRGs) in 2009 and returned to a global budget system in 2012. Changes in patient and hospital outcomes associated with moving from a DRG-based system to a global budget system for inpatient care have rarely been evaluated.

**Objective:**

To examine associations between the introduction and discontinuation of DRGs and changes in length of stay, volume of care, in-hospital mortality rates, and emergency readmission rates in the inpatient population in acute care hospitals overall, stratified by age group, and across 5 medical conditions.

**Design, Setting, and Participants:**

This cross-sectional study included data from patients aged 45 years or older who were hospitalized in public acute care settings in Hong Kong before the introduction (April 2006 to March 2009), during implementation (April 2009 to March 2012), and after discontinuation (April 2012 to November 2014) of the DRG scheme. Data analysis was conducted from January to June 2021.

**Exposures:**

Public hospitals transitioned from a global budget payment system to a DRG-based system in April 2009 and returned to a global budget system in April 2014.

**Main Outcomes and Measures:**

The main outcome was the association of use of DRGs with patient-level length of stay, in-hospital mortality rate, 1-month emergency readmission rate, and population-level number of admissions per month. An interrupted time series design was used to estimate changes in the level and slope of outcome variables after introduction and discontinuation of DRGs, accounting for pretrends.

**Results:**

This study included 7 604 390 patient episodes. Overall, the mean (SD) age of patients was 68.97 (13.20) years, and 52.17% were male. The introduction of DRGs was associated with a 1.77% (95% CI, 1.23%-2.32%) decrease in the mean length of stay, a 2.90% (95% CI, 2.52%-3.28%) increase in the number of patients admitted, a 4.12% (95% CI, 1.89%-6.35%) reduction in in-hospital mortality, and a 2.37% (95% CI, 1.28%-3.46%) decrease in emergency readmissions. Discontinuation of the DRG scheme was associated with a 0.93% (95% CI, 0.42%-1.44%) increase in the mean length of stay and a 1.82% (95% CI, 1.47%-2.17%) reduction in the number of patients treated after adjusting for covariates; no statistically significant change was observed in in-hospital mortality (−0.14%; 95% CI, −2.29% to 2.01%) or emergency readmission rate (−0.29%; 95% CI, −1.30% to 0.71%).

**Conclusions and Relevance:**

In this cross-sectional study, the introduction of DRGs was associated with shorter lengths of stay and increased hospital volume, and discontinuation was associated with longer lengths of stay and decreased hospital volume. In-hospital mortality and emergency readmission rates did not significantly change after discontinuation of DRGs.

## Key Points

**Question:**

Were the introduction and discontinuation of diagnosis-related groups (DRGs) for resource allocation in Hong Kong public hospitals associated with patients’ length of stay, hospitals’ volume of care, inpatient mortality rates, and emergency readmission rates?

**Findings:**

In this cross-sectional study of 7 604 390 patient episodes, the introduction of DRGs was associated with shorter hospital stays and increased hospital volume, and discontinuation was associated with longer stays and decreased volume. In-hospital mortality and emergency readmission rates decreased after introduction of DRGs but remained steady after their discontinuation.

**Meaning:**

In this study, the discontinuation of DRGs was associated with longer hospital stays and fewer hospital admissions but with no change in quality indicators.

## Teaser

This cross-sectional study examines associations between the introduction and discontinuation of use of diagnosis-related groups and length of stay, volume of care, in-hospital mortality, and emergency readmission rates among patients in public acute care hospitals in Hong Kong.

## Introduction

A diagnosis-related group (DRG) system is a patient classification system that categorizes clinical cases according to the total resources used for treatment.^1^ By condensing numerous cases into a manageable number of clinically meaningful and economically homogeneous groups, DRGs describe hospitals’ activities in standardized units and enable comparisons between them.^2^ In 1983, the US Medicare system became the first to adopt DRGs as the basis for paying hospitals. Other health care systems have since adopted DRGs to improve transparency or efficiency.^3^

The introduction of DRGs has been widely associated with changes in key outcomes that potentially affect patients and health care systems.^4,5,6,7,8^ Reductions in length of stay have generally been observed after the introduction of DRGs, whereas evidence on volume of care and other quality indicators is mixed.^2,6,9,10^ Some studies have attributed variations in the consequences of DRGs to health care system factors. Busse et al^2^ suggested that DRGs were associated with an increase in volume of care when replacing a global budget system but with a decrease when replacing a fee-for-service system. Vuagnat et al^10^ documented increased readmission rates at private hospitals but not public hospitals after the implementation of DRGs. More robust estimations are needed worldwide to determine the circumstances in which DRG-based systems are likely to produce positive or negative outcomes.

In this study, we examined changes associated with the implementation and discontinuation of a DRG-based resource allocation system in Hong Kong’s public hospitals.^11^ Managed by the Hospital Authority, Hong Kong’s public hospitals provide more than 90% of its citizens’ hospital services and rely almost entirely (ie, >90%) on an annual subvention from the government to finance health care delivery.^12^ The Hospital Authority’s system for allocating funding to hospitals has changed several times in recent years.^11^ Before 2009, the Hospital Authority used a population-based global budget approach, which was criticized for lack of transparency and incentives to improve efficiency and quality.^11,13^ This approach was supplanted by a pay-for-performance concept in 2009, which used DRGs to measure each hospital’s acute inpatient care volume and to adjust budgets across hospital clusters based on DRG-adjusted volume or weighted episodes. The Hospital Authority discontinued this scheme and reinstated a global budget approach in 2012.^11^

From 2009 to 2012, the Hospital Authority introduced an international refined DRG system that classified all possible combinations of more than 20 000 diseases and related treatments and procedures included in the *International Classification of Diseases, Ninth Revision, Clinical Modification* (*ICD-9-CM*) into more than 1000 groups with 3 levels of severity.^11,13^ This system was used to adjust budgets across public hospitals, measure hospitals’ performance, and allocate funding for service growth in targeted areas.^14^ The Hospital Authority reduced budgets for hospitals with actual costs higher than the DRG-adjusted cost and increased budgets for those with actual costs that were lower.^14^ This system replaced a global budget payment system that allocated resources to public hospitals according to their service targets at the start of each financial year. After 2012, the Hospital Authority discontinued use of DRGs and reinstated the global budget system.

As the first study, to our knowledge, of DRGs in Hong Kong, the current study supplements existing evidence on the implementation of DRGs in public hospitals in a tax-based system. Hong Kong’s experience in discontinuing a DRG-based payment scheme also provided a unique opportunity for analysis, allowing us to supplement the evidence on the consequences of its introduction. We examined whether the introduction and discontinuation of DRGs were associated with changes in length of stay, volume of care, emergency readmission rates, and in-hospital mortality.

## Methods

### Data Source

This cross-sectional study was part of a broader project on health care for older people that permitted our access to a patient-level administrative data set from public hospitals. The data set contains the clinical records of all patients aged 45 years or older who were hospitalized in an acute care setting from April 2006 to December 2014. The data set contains information on individual characteristics, diagnosis (*ICD-9-CM* codes), and care use. Patients have unique pseudo-identification numbers and sequence numbers for each of their episodes to document their flow through hospitals. The Survey and Behavioral Research Ethics Committee of the Chinese University of Hong Kong approved this study and waived requirement for informed patient consent owing to the use of deidentified data. The study followed the Strengthening the Reporting of Observational Studies in Epidemiology (STROBE) reporting guidelines for cross-sectional studies.

For volume of care, we aggregated monthly totals of inpatients by age group (45-54 years, 55-64 years, and ≥65 years), sex, and district. We used age-sex-district population figures provided by the Census and Statistics Department to calculate the number of admissions per 1000 residents per month.

### Outcomes

Outcome variables included length of stay, number of admissions per month, in-hospital mortality, and 1-month emergency readmission rates as proxies for quality of care. Length of stay was defined as the number of days spent during the index hospitalization. One-month emergency readmissions included readmissions to a hospital through the emergency department for any reason within 1 month of discharge from the index hospitalization. Index hospitalizations included all admissions through emergency or outpatient departments with patients discharged alive.

### Covariates

Covariates included individual demographic characteristics (age and sex), socioeconomic characteristics (nursing home status, social security assistance status, and median income of district of residence), year and month of admission, hospital cluster, and Charlson comorbidity index (CCI) calculated based on the primary diagnosis.^15^ In Hong Kong, people living in elder care residences tend to have poor health status^16,17^ and finances.^16^ We used harmonic terms to control for seasonality.^18,19^

### Statistical Analysis

We estimated descriptive statistics for patient characteristics and outcome variables for all hospitalizations during 3 phases: before introduction of the DRG scheme (April 2006 to March 2009), during implementation (April 2009 to March 2012), and after discontinuation (April 2012 to November 2014). We then used an interrupted time series design to estimate changes in the level and slope of outcome variables after the introduction and discontinuation of the DRG scheme, accounting for pretrends.^20^ Previous research suggests that hospitals may react to the introduction of a DRG scheme immediately and then adapt to it gradually.^4^ Therefore, we hypothesized that after the introduction of the DRG scheme, there would be both a level change (an immediate change in outcome variables) and a slope change (a gradual change in the trend’s gradient).^19^

We used a generalized linear regression with log link to specify the model for the outcome variables. Marginal effects were estimated to quantify percentage changes in outcome variables before and after the policy change. In the patient-level analyses for length of stay, in-hospital mortality, and emergency readmission, we included age, sex, social security assistance status, nursing home status, year and month of admission, hospital cluster, and CCI as covariates. In the population-level analysis for volume of care, adjustment variables included age, sex, district, year and month of admission, and population size. All tests were 2-sided, and *P* < .05 was considered significant.

We conducted subgroup analyses to examine whether changes in outcomes varied by age group or medical condition. Subgroups included elderly patients (aged ≥65 years) and nonelderly patients (aged 45-64 years) as well as patients with 5 primary diagnoses: congestive heart failure (*ICD-9-CM* code 428), acute myocardial infarction (*ICD-9-CM* codes 410 and 412), pneumonia (*ICD-9-CM* code 486), cerebrovascular diseases (*ICD-9-CM* codes 430-438), and hip fracture (*ICD-9-CM* code 820).^21^

To examine the robustness of our estimates, we conducted 3 sensitivity analyses. First, because any meaningful change to clinical practice in response to the new payment model would require time to develop, we excluded observations made immediately after the date of the policy implementation.^22^ Second, we calculated volume of care at the mega-cluster level, combining Hong Kong’s 18 districts into 3 geographic groups—Hong Kong Island, Kowloon, and New Territories—to mitigate the cross-district movement of patients. Third, we checked the sensitivity of our results to different measurements of disease burden first by calculating the CCI using patients’ primary and secondary diagnoses and then by replacing the index with a binary variable indicating whether the patient had a chronic disease. We used Stata software, version 14.0 (StataCorp) for all statistical analyses. Analyses were conducted from January to June 2021.

## Results

This study included 7 604 390 patient episodes. The mean (SD) age of patients was 68.97 (13.20) years, and 52.17% were male. Before the introduction of DRGs (April 2006 to March 2009), the mean (SD) age of patients was 69.04 (13.12) years, and 52.82% of patients were male. Patients receiving social security assistance and those living in nursing care homes accounted for 24.59% and 12.82% of the 2 187 767 hospitalizations, respectively. The mean (SD) CCI was 1.99 (0.92). The mean (SD) length of stay was 3.79 (4.62) days, and the mean (SD) number of hospital admissions per 1000 residents was 24.62 (17.73) per month. The in-hospital mortality rate was 3.06%, and the emergency readmission rate was 12.33%. Patient characteristics and outcome measures during implementation (April 2009 to March 2012) and after discontinuation (April 2012 to December 2014) of DRGs are shown in Table 1.

**Table 1.** Descriptive Statistics of Hospitalized Patient Characteristics and Outcome Variables Before, During, and After Implementation of DRGs^a^

Characteristic	Patients, No. (%)		
	Before DRGs (n = 2 187 767)	During DRGs (n = 2 718 599)	After DRGs (n = 2 698 024)
Age, mean (SD), y	69.04 (13.12)	68.98 (13.25)	68.88 (13.22)
Sex			
Female	1 032 608 (47.20)	1 298 531 (47.76)	1 306 418 (48.42)
Male	1 155 159 (52.82)	1 420 068 (52.24)	1 391 606 (51.58)
Received social security assistance	538 053 (24.59)	634 139 (23.33)	588 260 (21.78)
Lived in elder care residence	280 482 (12.82)	330 127 (12.14)	296 138 (10.98)
Household income, median (SD), HKD 1000^b^	18.01 (3.64)	20.81 (5.20)	27.51 (5.28)
Charlson comorbidity index score, mean (SD)	1.99 (0.92)	2.04 (0.87)	2.10 (0.86)
Outcome variables			
Length of stay, mean (SD), d	3.79 (4.62)	3.34 (4.31)	3.22 (4.24)
Hospital admissions, mean (SD), per 1000 residents per month	24.62 (17.73)	27.71 (19.65)	26.32 (19.06)
In-hospital deaths	66 889 (3.06)	72 054 (2.65)	66 224 (2.45)
1-mo Emergency readmissions	269 008 (12.33)	333 081 (12.25)	316 761 (11.74)

Abbreviation: DRGs, diagnosis-related groups.

^a^ The period of study before the introduction of DRGs was April 2006 to March 2009; during implementation, April 2009 to March 2012; and after discontinuation, April 2012 to November 2014.

^b^ 1 HKD is equal to 0.13 USD.

The Figure presents changes in outcome variables during the 3 periods. Table 2 summarizes the associations of the introduction and discontinuation of DRGs with the outcome variables. After controlling for pretrends and covariates, the introduction of DRGs was associated with an immediate 1.77% (95% CI, 1.23%-2.32%) decrease in length of stay and a gradual increase of 0.12% (95% CI, 0.09%-0.15%) per month. The discontinuation of DRGs was associated with an immediate 0.93% (95% CI, 0.42%-1.44%) increase in length of stay and a gradual increase of 0.25% (95% CI, 0.22%-0.28%) per month.

**Figure.**
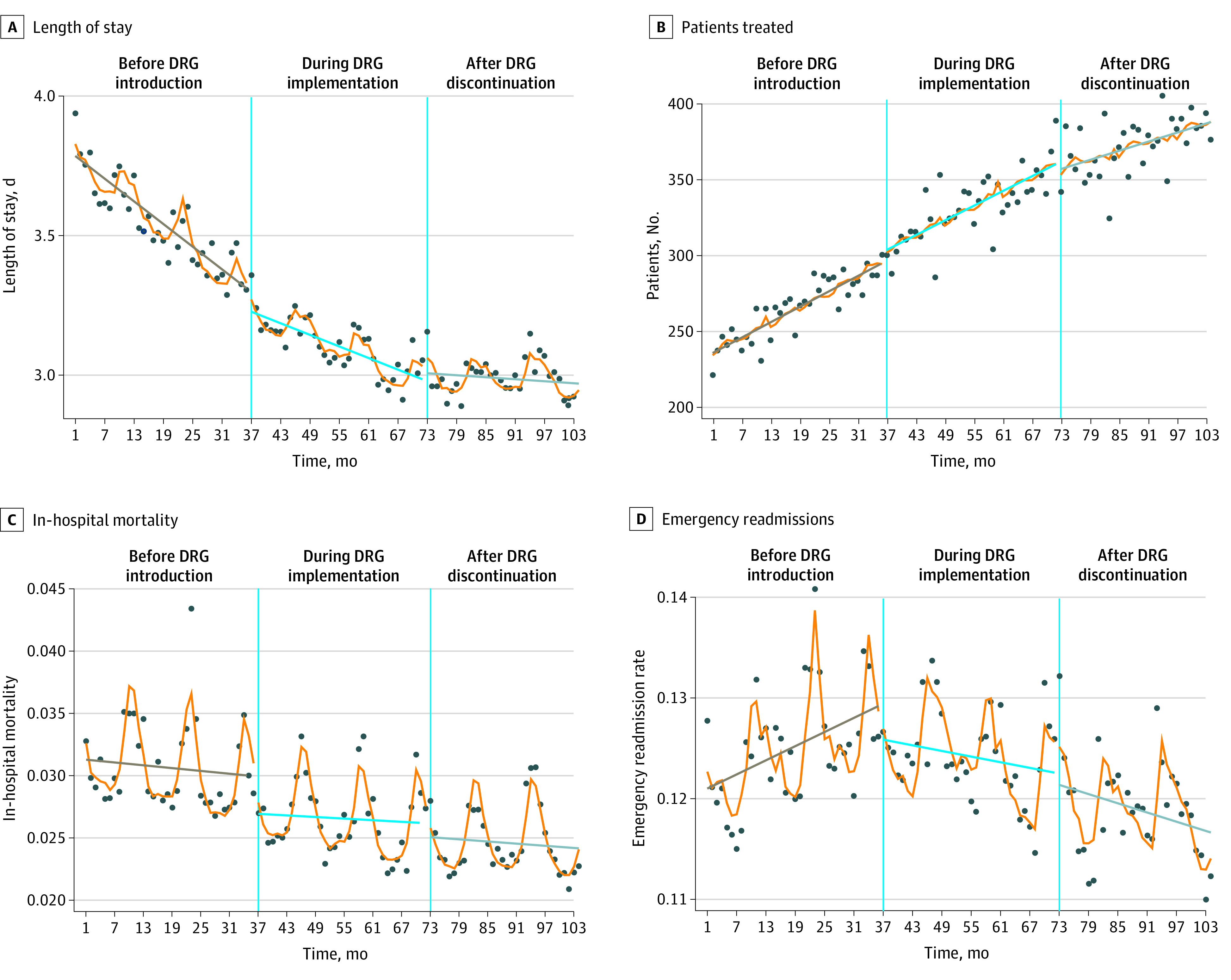
Changes in Outcome Variables Before, During, and After the Implementation of Diagnosis-Related Groups (DRGs) in Hong Kong Dots represent observed monthly mean of outcome variables; and slanted horizontal lines, linear fits of the predictions in the 3 phases.

**Table 2.** Changes in Outcome Variables After the Introduction and Discontinuation of DRGs^a^

Variable	After introduction of DRGs		After discontinuation of DRGs	
	Step change estimated coefficient (95% CI)	Slope change estimated coefficient (95% CI)	Step change estimated coefficient (95% CI)	Slope change estimated coefficient (95% CI)
Length of stay	−0.0177 (−0.0232 to −0.01228)^b^	0.0012 (0.0009 to 0.0015)^b^	0.0093 (0.0042 to 0.0144)^b^	0.0025 (0.0022 to 0.0028)^b^
Mean monthly admissions	0.0290 (0.0252 to 0.0328)^b^	−0.0018 (−0.0020 to −0.0016)^b^	−0.0182 (−0.0217 to −0.0147)^b^	−0.0025 (−0.0027 to −0.0024)^b^
In-hospital mortality	−0.0412 (−0.0635 to −0.0189)^b^	0.0002 (−0.0009 to 0.0014)	−0.0014 (−0.0229 to 0.0201)	0.0027 (0.0016 to 0.0038)^b^
Emergency readmission rate	−0.0237 (−0.0346 to −0.0128)^b^	−0.0016 (−0.0021 to −0.0011)^b^	−0.0029 (−0.0130 to 0.0071)	0.0006 (0.0001 to 0.0011)^c^

Abbreviation: DRGs, diagnosis-related groups.

^a^ All estimated coefficients were generated from generalized linear regression with log link. The estimated coefficients were directly interpreted as marginal effects. For example, an estimated coefficient of −0.0182 implied a 1.82% immediate decrease in length of stay associated with the introduction of DRGs.

^b^ *P* < .001.

^c^ *P* < .01.

Before the introduction of DRGs, the number of patients treated each month was increasing (Figure). The introduction of DRGs was associated with an immediate 2.90% (95% CI, 2.52%-3.28%) increase in monthly volume of care and a gradual decrease by 0.18% (95% CI, 0.16%-0.20%) per month. Discontinuation of DRGs was associated with an immediate 1.82% (95% CI, 1.47%-2.17%) reduction in monthly admissions and a gradual decrease of 0.25% (95% CI, 0.24%-0.27%) per month.

The introduction of DRGs was associated with a 4.12% (95% CI, 1.89%-6.35%) decrease in in-hospital mortality rates but no change in slope. Discontinuation of DRGs was not significantly associated with an immediate change in in-hospital mortality rates (−0.14%; 95% CI, −2.29% to 2.01%) but was associated with a 0.27% (95% CI, 0.16%-0.38%) increase in the slope of change.

We found a statistically significant increase in slope in emergency readmission rates in the period before the DRG scheme was implemented (Figure). The introduction of DRGs was associated with an immediate 2.37% (95% CI, 1.28%-3.46%) reduction in emergency readmissions and a gradual reduction of 0.16% (95% CI, 0.11%-0.21%) per month. Discontinuation of DRGs was not significantly associated with immediate changes in unplanned readmissions (−0.29%; 95%CI, −1.30% to 0.71%) but was associated with a gradual increase of 0.06% (95% CI, 0.01%-0.11%) per month.

eTables 1 and 2 in the Supplement present the subgroup analysis results. Outcomes among elderly and nonelderly patients were consistent with those in the total sample and were comparable to each other. We found that the introduction of DRGs was associated with a reduction in length of stay in all disease groups and with decreased in-hospital mortality in the groups with congestive heart failure, acute myocardial infarction, and pneumonia. Discontinuation of the DRG scheme was associated with an immediate increase in length of stay for patients with pneumonia and cerebrovascular disease and a gradual increase in length of stay for patients in all disease groups. All the sensitivity analyses confirmed the statistically significant associations identified in the main analysis (eTables 3-5 in the Supplement).

## Discussion

In this cross-sectional study, the introduction of the DRG scheme was associated with a decrease in length of stay and an increase in volume of care, and discontinuation was associated with longer lengths of stay and decreased hospital volume. Decreases in in-hospital mortality and emergency readmission rates were associated with the introduction of DRGs, but no significant change was associated with their discontinuation. These findings suggest that improvements in quality indicators after the introduction of DRGs might not have been associated with DRGs but may have been associated with other concurrent quality initiatives.

Before the introduction of DRGs, public hospitals in Hong Kong were pressured to reduce length of stay because of increasing congestion in hospital wards and the government’s Enhanced Productivity Programme.^11,23,24^ The introduction of DRGs appeared to be associated with accelerated reductions in length of stay, consistent with evidence from the US and European countries.^4,5,21,25^ The standardized price per DRG group incentivizes hospitals to generate a surplus by reducing unit costs (eg, by reducing length of stay).^7^

Volume of care increased significantly in association with the introduction of DRGs, consistent with evidence from most European countries that have moved to DRG-based payments from global budget systems.^5,25^ Global budget systems limit total expenditures and eliminate the need for increased production,^2,11,26^ whereas retrospective payment systems based on DRGs link hospitals’ payments to the number of patients they treat, incentivizing them to treat more patients.^25^ However, in the US, where DRGs succeeded a fee-for-service system, hospital activity generally decreased.^4^ Our findings thus echo previous arguments that contextual factors, such as the prior payment system, may largely determine the effects of DRGs.^2^

Discontinuation of DRGs was associated with an increase in length of stay and a decrease in the number of patients treated, reversing the trends that followed the program’s introduction. These findings suggest that the reintroduction of a global budget removed incentives for hospitals to increase throughput by reducing cost per patient, providing further evidence that the changes in length of stay and volume of care in 2009 may have been associated with the introduction of DRGs. However, the magnitude of change after the discontinuation of DRGs was smaller than that associated with their introduction. One explanation could be the overall increase in the Hospital Authority’s budget, which may have contributed to the changes in 2009.^24,27^ Similar to Hong Kong, a DRG-based system was replaced with a global budget payment system in Maryland in 2014. Consistent with our findings, some studies in Maryland found that the change was associated with statistically significant decreases in length of stay and hospital admissions,^28,29,30^ although others found that the decreases were not significant.^30,31,32^

The introduction of DRGs was also associated with significant decreases in emergency readmission and in-hospital mortality rates, consistent with US-based research documenting a decrease in the number of inpatient deaths after implementation of DRGs.^9^ This decrease was accompanied by increases in deaths in nursing homes, suggesting that hospitals attempted to control costs by transferring terminally ill patients to alternative settings.^33^ Our lack of data on deaths outside acute care hospital settings precluded us from examining changes in place of death. However, given the lack of changes in legal and economic factors that would discourage deaths at hospitals in Hong Kong during the study period, it seems unlikely that shifts in place of death would explain the sudden decrease in mortality rates.^17^ Outside the US, previous research has rarely identified changes in readmissions or mortality after the introduction of DRGs.^6,9,25,34,35^

We found that discontinuation of DRGs was not associated with changes in readmission or mortality rates, consistent with the findings of research conducted in Maryland.^28,30,31,32,36,37^ These findings further suggest that the changes in quality indicators after the introduction of DRGs were associated with other concurrent quality initiatives in the Hospital Authority, such as the Key Performance Indicator framework,^11^ the Quality Incentive Programme,^11,38^ and the Surgical Outcomes Monitoring and Improvement Programme.^39^ In our subgroup analyses, we chose diseases with high mortality and process-outcome associations for which the decline in quality of care would likely manifest in outcome changes.^21,40^ We did not find increased mortality or emergency readmissions after the introduction of DRGs and even found an improvement in quality indicators for some conditions. Specifically, congestive heart failure and acute myocardial infarction were 2 of the diseases under special surveillance in the Key Performance Indicator framework introduced in 2010,^41^ which may explain the relatively higher reduction in hospital mortality in these subgroups compared with the subgroups with other diseases. Our subgroup analysis also showed comparable results between elderly and nonelderly groups; it seems unlikely that health care programs targeting elderly patients introduced in 2008 and 2009 would explain improvements in quality indicators in the whole sample.^42^

Although no decrements in quality of care were observed in association with DRGs, other issues in hospitals after the introduction of the DRG-based system contributed to its discontinuation. First, the standardized rate per case led to loss of hospitals for which costs were high compared with standardized rates^4^—typically large hospitals in which complex cases were treated. The complaints from the chief executives of large hospitals who were usually part of the Hospital Authority’s senior management team contributed to the end of DRGs.^11^ Second, increased productivity without compromised quality of care was achieved through increased workloads for health care professionals. Given long-standing labor shortage issues in Hong Kong’s health care system,^43^ the Hospital Authority terminated the DRG program to avoid increasing turnover among public health care professionals.

### Limitations

This study has several limitations. First, Hong Kong implemented DRGs for only 3 years; thus, we could not observe their long-term effects. Previous research suggests that their effects tend to be greatest during the initial implementation period and stabilize or dissipate over time.^4^ Research has documented increased readmissions in Switzerland^44^ and France,^10^ suggesting that more research on the long-term association of DRGs with quality of care is warranted. Second, our data only included readmissions and deaths in public hospitals; readmissions to private hospitals and deaths outside public hospitals were not captured. Nevertheless, public hospitals dominate inpatient care provision, especially for patients with severe conditions, and most deaths occur in hospitals rather than in homes or other institutions.^17^ No changes in contextual factors that might have been associated with shifts in inpatient admissions and deaths and confounded the outcomes were reported during the study period. Third, our study lacked a control group, limiting our ability to rule out confounding factors. Nevertheless, if assuming that going back to a global budget system after discontinuation of DRGs would reverse outcome trends to which it contributed, our estimation of the changes after discontinuation of DRGs strengthens our confidence that the increase in volume of care and the reduction in length of stay were associated with the DRG scheme, whereas changes in quality indicators were likely associated with other initiatives. Fourth, our study was part of a broader project on health care for older people, which limited our access to the clinical records of patients aged 45 years or older in public hospitals and prevented us from extending our findings to younger patients. Nevertheless, this age group accounts for approximately 70% of public inpatient admissions in Hong Kong,^45^ and one would expect to see the same pattern for all admissions as in the analyzed sample.

## Conclusions

In this cross-sectional study, the introduction and discontinuation of DRGs were associated with opposite changes in length of stay and volume of care in public hospitals in Hong Kong. Decreased in-hospital mortality and emergency readmission rates were associated with the introduction of DRGs, but no significant changes in these outcome measures were associated with discontinuation of DRGs. Further study is warranted to evaluate the effects of DRGs on quality of care while ruling out confounding factors.

## Notes

Supplement. **eTable 1.** Changes in Outcome Variables Associated With the Introduction and Discontinuation of DRGs by Age Group **eTable 2.** Changes in Outcome Variables Associated With the Introduction and Discontinuation of DRGs by Disease Group **eTable 3.** Changes in Outcome Variables Associated With the Introduction and Discontinuation of DRGs, Excluding Transition Periods After Policy Changes **eTable 4.** Changes in Outcome Variables Associated With the Introduction and Discontinuation of DRGs, Using Alternative Approaches to Estimate Patients’ Disease Burden **eTable 5.** Changes in Volume of Care Associated With the Introduction and Discontinuation of DRGs, Population Number of Admissions on Mega-Cluster Level
