# Supplementary material for: Evaluation of Length of Stay, Care Volume, In-Hospital Mortality, and Emergency Readmission Rate Associated With Use of Diagnosis-Related Groups for Internal Resource Allocation in Public Hospitals in Hong Kong
Source: JAMA Netw Open. 2022 Feb 4;5(2):e2145685. doi: 10.1001/jamanetworkopen.2021.45685 (PMC8817200; doi:10.1001/jamanetworkopen.2021.45685)
Supplement: Supplement. — eTable 1. Changes in Outcome Variables Associated With the Introduction and Discontinuation of DRGs by Age Group eTable 2. Changes in Outcome Variables Associated With the Introduction and Discontinuation of DRGs by Disease Group eTable 3. Changes in Outcome Variables Associated With the Introduction and Discontinuation of DRGs, Excluding Transition Periods After Policy Changes eTable 4. Changes in Outcome Variables Associated With the Introduction and Discontinuation of DRGs, Using Alternative Approaches to Estimate Patients’ Disease Burden eTable 5. Changes in Volume of Care Associated With the Introduction and Discontinuation of DRGs, Population Number of Admissions on Mega-Cluster Level [file jamanetwopen-e2145685-s001.pdf]

Supplementary Online Content

Wu Y, Fung H, Shum HM, et al. Evaluation of length of stay, care volume, in-hospital mortality, and emergency readmission rate associated with use of diagnosis-related groups for internal resource allocation in public hospitals in Hong Kong. *JAMA Netw Open*. 2022;5(2):e2145685. doi:10.1001/jamanetworkopen.2021.45685

eTable 1. Changes in Outcome Variables Associated With the Introduction and Discontinuation of DRGs by Age Group
eTable 2. Changes in Outcome Variables Associated With the Introduction and Discontinuation of DRGs by Disease Group
eTable 3. Changes in Outcome Variables Associated With the Introduction and Discontinuation of DRGs, Excluding Transition Periods After Policy Changes
eTable 4. Changes in Outcome Variables Associated With the Introduction and Discontinuation of DRGs, Using Alternative Approaches to Estimate Patients' Disease Burden
eTable 5. Changes in Volume of Care Associated With the Introduction and Discontinuation of DRGs, Population Number of Admissions on Mega-Cluster Level

This supplementary material has been provided by the authors to give readers additional information about their work.

© 2022 Wu Y et al. *JAMA Network Open*.

Supplement

eTable 1. Changes in outcome variables associated with the introduction and discontinuation of DRGs by age group

	Introduction of DRGs		Discontinuation of DRGs	
	Step change estimated coefficients [95%CI]	Slope change estimated coefficients [95%CI]	Step change estimated coefficients [95%CI]	Slope change estimated coefficients [95%CI]
**Patients aged 45–64 years**				
Length of stay	-0.0271**	0.0012 ***	0.0139**	0.0019***
	[-0.0364, -0.0177]	[0.007,0.0017]	[0.0054, 0.0224]	[0.0014, 0.0024]
Average monthly admissions per month	0.0343***	-0.0007***	-0.0219***	-0.0018***
	[0.0283, 0.0402]	[-0.0010, -0.0004]	[-0.0273, -0.0165]	[-0.0021, -0.0016]
In-hospital mortality (%)	-0.1076***	-0.0005	-0.0068	0.0050***
	[-0.1636, -0.0515]	[-0.0033, 0.0023]	[-0.0611, 0.0475]	[0.0021, 0.0078]
Emergency readmission (%)	-0.0549***	-0.0021***	0.0003	0.0004
	[-0.0785, -0.0314]	[-0.0032, -0.0009]	[-0.0210, 0.0216]	[-0.0007, 0.0015]
**Patients aged 65+ years**				
Length of stay	-0.0105***	0.0010***	0.0066***	0.0030***
	[-0.0172, -0.0039]	[0.0006, 0.0013]	[0.0003, 0.0129]	[0.0027, 0.0034]
Average monthly admissions per month	0.0258***	-0.0025***	-0.0161***	-0.0031***
	[0.0209, 0.0307]	[-0.0028, -0.0023]	[-0.0207, -0.0115]	[-0.0034, -0.0029]
In-hospital mortality (%)	-0.0295*	0.0002	0.0012	0.0024***
	[-0.0538, -0.0053]	[-0.0010, 0.0014]	[-0.0223, 0.0247]	[0.0011, 0.0036]
Emergency readmission (%)	-0.0170**	-0.0017***	-0.0044	-0.0002
	[-0.0293, -0.0047]	[-0.0023, -0.0011]	[-0.0159, 0.0071]	[-0.0008, 0.0004]

*** $P < .001$; ** $P < .01$; * $P < .05$.

© 2022 Wu Y et al. *JAMA Network Open*.

eTable 2. Changes in outcome variables associated with the introduction and discontinuation of DRGs by disease group

	Introduction of DRGs		Discontinuation of DRGs	
	Step change estimated coefficients [95%CI]	Slope change estimated coefficients [95%CI]	Step change estimated coefficients [95%CI]	Slope change estimated coefficients [95%CI]
**Congestive heart failure**				
Length of stay	-0.0267*	0.0014*	-0.0181	0.0025**
	[-0.0528, -0.0007]	[0.0000,0.0028]	[-0.0136, 0.0498]	[0.0009, 0.0042]
In-hospital mortality (%)	-0.1934**	-0.0015	0.0472	0.0009
	[-0.3342, -0.0527]	[-0.0087, 0.0056]	[-0.1006, 0.1951]	[-0.0069, 0.0087]
Emergency readmission (%)	-0.0101	-0.0015	-0.0373	0.0012
	[-0.0617, 0.0416]	[-0.0041, 0.0011]	[-0.0886, 0.0140]	[-0.0015, 0.0039]
**Acute myocardial infarction**				
Length of stay	-0.0858***	-0.0025***	0.0050	0.0051***
	[-0.1248, -0.0467]	[-0.0044, -0.0006]	[-0.0290, 0.0390]	[0.0033, 0.0070]
In-hospital mortality (%)	-0.2107***	-0.0048	-0.1305**	0.0062*
	[-0.3177, -0.1037]	[-0.0100, 0.0005]	[-0.2273, -0.0338]	[0.0011, 0.0113]
Emergency readmission (%)	-0.0279	-0.0033	0.0331	-0.0022
	[-0.1577, 0.1019]	[-0.0095, 0.0030]	[-0.0649, 0.1311]	[-0.0074, 0.0030]
**Pneumonia**				
Length of stay	-0.0176	0.0018***	0.0011	0.0036***
	[-0.0384, 0.0032]	[-0.0028, -0.0007]	[-0.0257, 0.0279]	[0.0022, 0.0050]
In-hospital mortality (%)	-0.0614*	-0.0003	0.0429	0.0061***
	[-0.1119, -0.0109]	[-0.0029, 0.0022]	[-0.0037, 0.0895]	[0.0036, 0.0085]
Emergency readmission (%)	0.0421	-0.0015	0.0114	0.0005
	[-0.0053, 0.0895]	[-0.0039, 0.0009]	[-0.0322, 0.0550]	[-0.0018, 0.0028]
**Cerebrovascular disease**				
Length of stay	-0.1265***	-0.0025***	0.0066*	0.0030***
	[-0.1649, -0.0882]	[-0.0045, -0.0005]	[0.0003, 0.0129]	[0.0027, 0.0034]
In-hospital mortality (%)	-0.0263	-0.0040	0.0200	0.0081***
	[-0.1073, 0.0547]	[-0.0081, 0.0000]	[-0.0645, 0.1045]	[0.0037, 0.0125]
Emergency readmission (%)	-0.0421	-0.0064**	0.0062	0.0005
	[-0.1254, 0.0411]	[-0.0106, -0.0022]	[-0.0763, 0.0886]	[-0.0038, 0.0048]
**Hip fracture**				
Length of stay	-0.0170*	-0.0002	0.0352***	0.0044***
	[-0.0429, -0.0089]	[-0.0011, 0.0015]	[0.0161, 0.0543]	[0.0033, 0.0054]
In-hospital mortality (%)	0.0964	-0.0118	0.0927	0.0154
	[-0.2022, 0.3949]	[-0.0270, 0.0034]	[-0.2474, 0.4328]	[-0.0019, 0.0328]
Emergency readmission (%)	-0.1054	-0.0028	0.0210	0.0016
	[-0.2982, 0.0875]	[-0.0125, 0.0069]	[-0.1654, 0.2074]	[-0.0082, 0.0113]

*** *P* < .001; ** *P* < .01; * *P* < .05.

© 2022 Wu Y et al. *JAMA Network Open*.

eTable 3. Changes in outcome variables associated with the introduction and discontinuation of DRGs, excluding transition periods after policy changes

	Introduction of DRGs		Discontinuation of DRGs	
	Step change estimated coefficients [95%CI]	Slope change estimated coefficients [95%CI]	Step change estimated coefficients [95%CI]	Slope change estimated coefficients [95%CI]
**Transition period: 6 months**				
Length of stay	-0.0255***	0.0013***	0.0104**	0.0026***
	[-0.0321, -0.0189]	[0.0010, 0.0017]	[0.0040, 0.0168]	[0.0023, 0.0030]
Average monthly admissions per month	0.0418***	-0.0024***	-0.0336***	-0.0017***
	[0.0374, 0.0463]	[-0.0026, -0.0022]	[-0.0380, -0.0292]	[-0.0019, -0.0015]
In-hospital mortality (%)	-0.0302*	-0.0003	-0.0254	0.0039***
	[-0.0561, -0.0043]	[-0.0016, 0.0010]	[-0.0518, 0.0010]	[0.0026, 0.0053]
Emergency readmission (%)	-0.0322***	-0.0012**	-0.0112	0.0003
	[-0.0483, -0.0160]	[-0.0021, -0.0004]	[-0.0269, 0.0045]	[-0.0005, 0.0012]
**Transition period: 3 months**				
Length of stay	-0.0223***	0.0012***	0.0086**	0.0027***
	[-0.0281, -0.0164]	[0.0009, 0.0015]	[0.0031, 0.0142]	[0.0024, 0.0030]
Average monthly admissions per month	0.0394***	-0.0023***	-0.0271***	-0.0020***
	[0.0354, 0.0435]	[-0.0025, -0.0021]	[-0.0309, -0.0233]	[-0.0022, -0.0018]
In-hospital mortality (%)	-0.0378**	0.0001	0.0004	0.0038***
	[-0.0612, -0.0143]	[-0.0011, 0.0012]	[-0.0004, 0.0011]	[0.0026, 0.0050]
Emergency readmission (%)	-0.0257***	-0.0015***	-0.0117	0.0004
	[-0.0402, -0.0112]	[-0.0023, -0.0007]	[-0.0254, 0.0021]	[-0.0004, 0.0011]
**Transition period: 1 month**				
Length of stay	-0.0218***	0.0012***	0.0069*	0.0028***
	[-0.0274, -0.0163]	[0.0009, 0.0015]	[0.0015, 0.0123]	[0.0025, 0.0031]
Average monthly admissions per month	0.0296***	-0.0018***	-0.0143***	-0.0027***
	[0.0258, 0.0335]	[-0.0020, -0.0017]	[-0.0180, -0.0107]	[-0.0029, -0.0025]
In-hospital mortality (%)	-0.0369**	0.0000	-0.0134	0.0032***
	[-0.0592, -0.0145]	[-0.0011, 0.0012]	[-0.0352, 0.0084]	[0.0021, 0.0044]
Emergency readmission (%)	-0.0235***	-0.0016***	-0.0113	0.0004
	[-0.0372, -0.0097]	[-0.0024, -0.0009]	[-0.0240, 0.0015]	[-0.0004, 0.0011]

Note: A transition period was considered by excluding observations immediately after the dates of the policy changes. For example, when considering a 6-month transition period, we excluded 6 months of data after the policy changes in April 2009 and in April 2012, respectively.
*** $P < .001$; ** $P < .01$; * $P < .05$.

© 2022 Wu Y et al. *JAMA Network Open*.

eTable 4. Changes in outcome variables associated with the introduction and discontinuation of DRGs, using alternative approaches to estimate patients' disease burden

	Introduction of DRGs		Discontinuation of DRGs	
	Step change estimated coefficients [95%CI]	Slope change estimated coefficients [95%CI]	Step change estimated coefficients [95%CI]	Slope change estimated coefficients [95%CI]
**Charlson comorbidity index based on primary and secondary diagnoses**				
Length of stay	-0.0162***	0.0017***	0.0076**	0.0025***
	[-0.0224, -0.0099]	[0.0013, 0.0021]	[0.0020, 0.0131]	[0.0021, 0.0028]
In-hospital mortality (%)	-0.0432***	0.0017**	0.0071	0.0024***
	[-0.0656, -0.0209]	[0.0005, 0.0029]	[-0.0143, 0.0286]	[0.0013, 0.0036]
Emergency readmission (%)	-0.0283***	-0.0010*	-0.0051	-0.0003
	[-0.0424, -0.0143]	[-0.0019, -0.0002]	[-0.0180, 0.0077]	[-0.0010, 0.0005]
**Whether the patient had a chronic disease**				
Length of stay	-0.0165***	0.0017***	0.0078**	0.0025**
	[-0.0229, -0.0101]	[0.0013, 0.0021]	[0.0022, 0.0135]	[0.0022, 0.0029]
In-hospital mortality (%)	-0.0198	0.0017**	0.0052	0.0025***
	[-0.0417, 0.0021]	[0.0006, 0.0028]	[-0.0160, 0.0264]	[0.0013, 0.0036]
Emergency readmission (%)	-0.0268***	-0.0009*	-0.0056	0.0005
	[-0.0409, -0.0127]	[-0.0017, -0.0000]	[-0.0185, 0.0073]	[-0.0003, 0.0013]

Note: In this sensitivity analysis, we used two alternative approaches to measuring disease burden rather than the Charlson comorbidity index based on primary diagnosis. First, we controlled for Charlson comorbidity index calculated based on both primary and secondary diagnoses. Second, we controlled for whether the patient had any chronic disease.

*** $P < .001$; ** $P < .01$; * $P < .05$.

eTable 5. Changes in volume of care associated with the introduction and discontinuation of DRGs, population number of admissions on mega-cluster level

	Introduction of DRGs		Discontinuation of DRGs	
	Step change estimated coefficients [95% CI]	Slope change estimated coefficients [95% CI]	Step change estimated coefficients [95% CI]	Slope change estimated coefficients [95% CI]
Average monthly admissions per month	0.0324***	-0.0021***	-0.0145***	-0.0020***
	[0.0284, 0.0364]	[-0.0023, -0.0019]	[-0.0182, -0.0108]	[-0.0022, -0.0019]

Note: The number of hospital admissions is counted at the mega-cluster level, which combines the existing 18 districts into 3 larger geographical groups—Hong Kong Island, Kowloon, and New Territories—to mitigate the cross-cluster movement of patients.

*** $P < .001$; ** $P < .01$; * $P < .05$.

© 2022 Wu Y et al. *JAMA Network Open*.
